# The Diagnosis and Treatment of Hæmorrhoids

**Published:** 1887-11

**Authors:** 


THE DIAGNOSIS AND TREATMENT OF HÆMORRHOIDS. By Chas. B. Kelsey, M. D., 1887. George S. Davis, Detroit. p.p. 78.

Clear, succinct, brief and practical, embracing chiefly the author's experience and observation in this line. Of course the subject is not exhausted. There is evidently no such intention. It includes, however, definite statements and directions which will aid any practitioner, and enable him to treat such cases more efficiently. In speaking of carbolic acid injections, the author tones down his former enthusiastic utterances. This he seeks to explain by claiming a wider and more unfortunate experience. But it is difficult to reconcile the following statements, unless by a too zealous advocacy in the first place, or the carelessness of routine in the second:

" The amount of sloughing has always been in direct proportion to the strength and quantity of injection, *can be regulated at will*, and has never been of sufficient extent to cause me the slightest mental uneasiness. In some cases I deliberately cause a slough each time I make an injection; in others I carefully avoid a slough. *Either can be done at will.*"*

On page 50 of the book now being noticed, the author says, "By following these rules all went well for a time, but soon I began to be troubled with a constant succession of sloughs with their attendant pain; and the worst of the trouble was that I *never knew when a slough was likely to be caused.* . . . So that after a time I was forced to confess that I had no means of determining beforehand whether the patient was to undergo the pain of an inflamed and sloughing hæmorrhoid."

We cannot but admit that it was right and proper for the writer to be "not consistent, but simply true." But how much better to adapt all generalizations to the facts and not attempt to anticipate unrealized possibilities.

The author has made no mention of the *ecraseur*, one of the most convenient means of treatment. True, to a specialist it must yield its claims before others, but to a general practitioner or surgeon, whose hæmorrhoidal cases are not legion, it presents many points of positive excellence. Not only is it as thorough, "safe and certain, and free from complications" as the clamp, but above all it is less liable to do harm in inexperienced though not unskillful hands. No method by operation is so easily acquired.

It may seem that so small a work should not need so extended a review; but the author treats of an affection more frequent and bothersome, not to be more dangerous to life, than almost any diseased condition. Measured by its usefulness and practical applicability, this little book becomes of great value. A clear, definite teaching cannot but benefit either in its action or reaction.

H. H.

---

*Am. Jour. Med. Sci., July, 1885, p. 176. (The italics are ours.)

